# Action prediction in psychosis

**DOI:** 10.1038/s41537-023-00429-x

**Published:** 2024-01-10

**Authors:** Noemi Montobbio, Enrico Zingarelli, Federica Folesani, Mariacarla Memeo, Enrico Croce, Andrea Cavallo, Luigi Grassi, Luciano Fadiga, Stefano Panzeri, Martino Belvederi Murri, Cristina Becchio

**Affiliations:** 1 Center for Human Technologies, Fondazione Istituto Italiano di Tecnologia, Via Enrico Melen 83, 16152 Genoa, Italy; 2 Department of Health Sciences (DISSAL), University of Genoa, Via A. Pastore 1, 16132 Genoa, Italy; 3 Institute of Psychiatry, Department of Neuroscience and Rehabilitation, University of Ferrara, Via Fossato di Mortara 64, 44121 Ferrara, Italy; 4 Dipartimento di Psicologia, Università di Torino, Via Giuseppe Verdi, 10, 10124 Torino, Italy; 5 Center for Translational Neurophysiology, Fondazione Istituto Italiano di Tecnologia, Via Fossato di Mortara 19, 44121 Ferrara, Italy; 6 Section of Physiology, Department of Neuroscience and Rehabilitation, University of Ferrara, Via Fossato di Mortara 19, 44121 Ferrara, Italy; 7 Institute of Neural Information Processing, Center for Molecular Neurobiology (ZMNH), University Medical Center Hamburg-Eppendorf (UKE), Falkenried 94, 20251 Hamburg, Germany; 8 Department of Neurology, University Medical Center Hamburg-Eppendorf (UKE), Martinistraße 52, 20246 Hamburg, Germany

**Keywords:** Human behaviour, Psychosis

## Abstract

Aberrant motor-sensory predictive functions have been linked to symptoms of psychosis, particularly reduced attenuation of self-generated sensations and misattribution of self-generated actions. Building on the parallels between prediction of self- and other-generated actions, this study aims to investigate whether individuals with psychosis also demonstrate abnormal perceptions and predictions of others’ actions. Patients with psychosis and matched controls completed a two-alternative object size discrimination task. In each trial, they observed reaching actions towards a small and a large object, with varying levels of temporal occlusion ranging from 10% to 80% of movement duration. Their task was to predict the size of the object that would be grasped. We employed a novel analytic approach to examine how object size information was encoded and read out across progressive levels of occlusion with single-trial resolution. Patients with psychosis exhibited an overall pattern of reduced and discontinuous evidence integration relative to controls, characterized by a period of null integration up to 20% of movement duration, during which they did not read any size information. Surprisingly, this drop in accuracy in the initial integration period was not accompanied by a reduction in confidence. Difficulties in action prediction were correlated with the severity of negative symptoms and impaired functioning in social relationships.

## Introduction

Complex, high-level dysfunctions are often grounded in subtle abnormalities in low-level processes. In the case of psychosis, theoretical considerations and empirical findings link psychotic symptoms, such as misattributions of self-generated actions to others and diminished demarcation of self-other boundaries, to abnormal sensorimotor predictions^1^.

When an action is performed, an internal forward model uses an efference copy of the motor commands sent to the muscles to predict the sensory outcome from that action^2^. This predicted outcome is then compared to the actual sensory outcome. If they match, the action sensory outcome is attenuated^3^. This process is believed to provide a mechanism to filter the sensory information that arises from our own actions and differentiate between self-generated and other-generated actions^3^. A growing body of research support the hypothesis that a failure in this mechanism may underlie the reduced sensory attenuation and the emergence of delusions of controls in psychosis^4^. Specifically, due to incorrect anticipations about the sensory feedback from their actions, individuals with psychosis may experience a mismatch between the predicted and actual sensory outcome^5–10^. This can lead to failure in sensory attenuation, resulting in the individual perceiving their own actions as surprising and externally controlled. Supporting this notion, abnormal sensorimotor predictions have been related to auditory misattributions in early psychosis^9^. Moreover, evidence of sensorimotor deficits underlying the sense of agency has been reported in individuals at increased risk of schizophrenia^8^.

These studies focused on self-generated actions, leaving a key question unanswered: do sensorimotor prediction deficits extend to others’ actions?^11^. This question is driven by the computational parallels between the processing of self-generated and other-generated actions^12^ and, more specifically, by the notion that internal forward models used to predict the sensory outcomes of self-generated actions are also utilized for predicting the outcomes of other-generated actions^13^. This generates the prediction that individuals with psychosis, characterized by aberrant internal forward models, would also exhibit abnormal perceptions and predictions of others’ actions. Contrary to this prediction, previous research did not find any abnormalities in action prediction among individuals with psychosis. For instance, Chambon et al.^14^, found that, in comparison to healthy controls, patients with schizophrenia encountered difficulties in inferring the intention of a sequence of manipulative actions (intention understanding), but not in discriminating the outcomes of individual actions (action prediction).

However, potential abnormalities at the action level could be concealed by the rapidity with which action information is processed. In a previous study utilizing a progressive temporal occlusion design task, we demonstrated that healthy observers have the ability to accurately predict the outcome of a manipulative action, specifically determining the size of an object to be grasped, by observing only 10% of the reach-to-grasp movement, which corresponds to ~80 ms^15^. The accuracy of size predictions improves rapidly over time, reaching near-perfect accuracy around 60% of the movement duration^15^. Previous studies in patients exclusively investigated predictions made from late occluded actions (with occlusions occurring between 79% and 100% of movement duration)^14^. Therefore, it remains unclear whether early integration processes crucial for rapid action predictions are intact among individuals with psychosis.

**Fig. 1:**
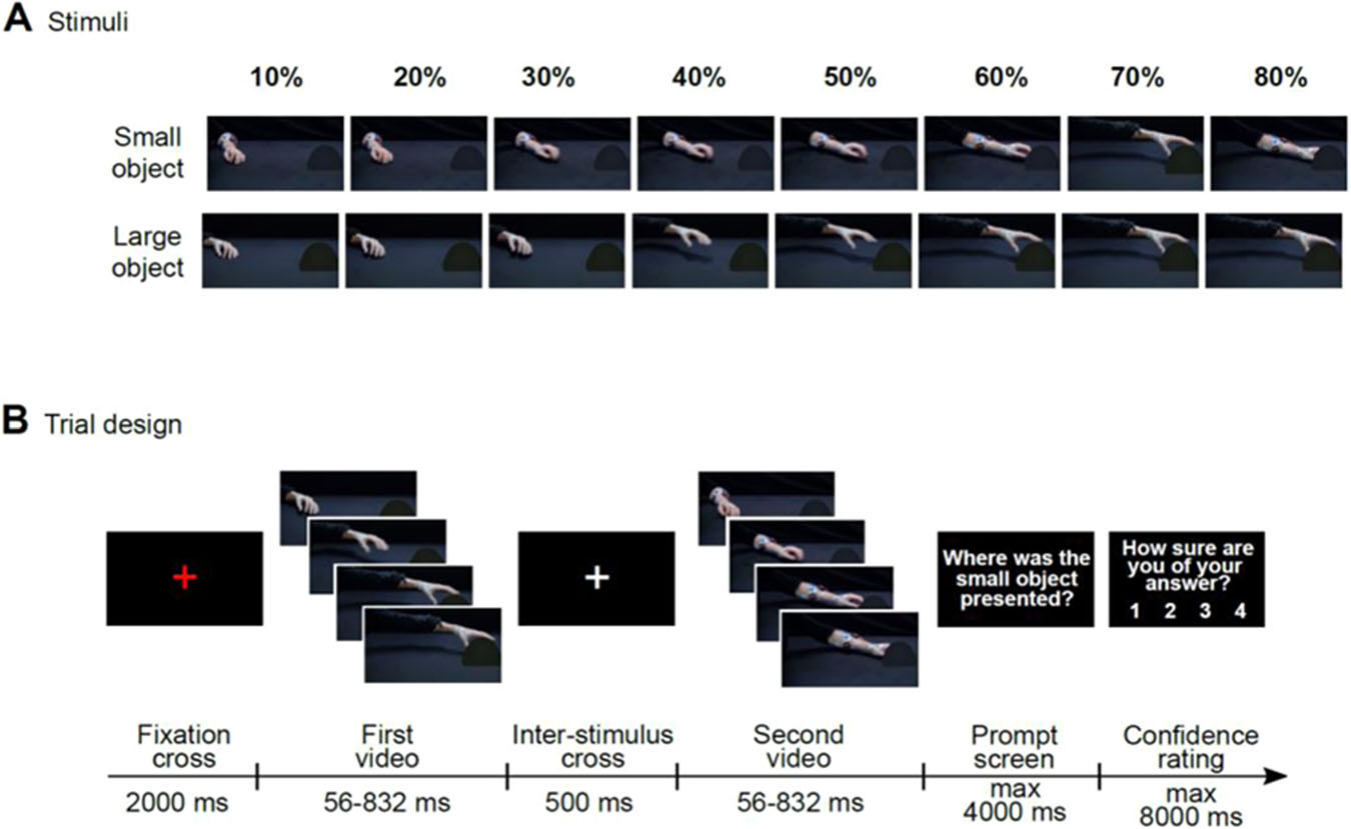
Experimental design. **A** Example video frames of reach-to-grasp actions towards a small or large object presented under eight levels of temporal occlusion. **B** Trial design of the action prediction task. For display purposes, frames are flipped over the vertical midline with respect to their original orientation.

To address this gap, we investigated the ability of patients with psychosis to predict the size of a to-be-grasped object over progressive temporal occlusion intervals (Fig. 1A) and used the recently developed kinematic coding framework^16–18^ to examine their ability to extract size information with single-trial resolution.

## Methods

### Participants

Sixteen outpatients (8 females) diagnosed with non-affective psychotic disorders (schizophrenia, schizoaffective disorder, and brief psychotic disorder) and 16 control participants (11 females) participated in the study. Controls were matched to patients according to age (*t*_(30)_ = −1.83, *p* = 0.077). Patients were recruited from the Community Mental Health Services in Ferrara and had previously been diagnosed by an experienced psychiatrist according to ICD-9 CM criteria (in use at the regional level for diagnosis classification). Patients with neurological disorders, comorbid major depression and/or substance abuse according to ICD-9 CM criteria were excluded. Psychiatric, neurological, and substance-use disorders were exclusion criteria for controls. All participants had normal or corrected-to-normal vision. Baseline characteristics of participants are reported in Supplementary Table 1. The study was approved by the “Area Vasta” Ethics Committee of the Region Emilia Romagna and complied with the principles of the revised Helsinki Declaration^19^. All participants received information about the content of the study and provided written informed consent prior to participation in the study. The sample size was determined according to a power calculation based on ref. ^15^ (see Supplementary Methods).

#### Assessment of symptomatology and functioning

Symptom severity, cognitive functioning and neurological status were assessed in the patient group using the Positive and Negative Syndrome Scale (PANSS)^20^, the Brief Psychiatric Rating Scale (BPRS)^21^, the Brief Negative Symptom Scale (BNSS)^22^, the Trail Making Test (TMT B-A)^23^, and the Neurological Evaluation Scale (NES)^24^. To measure real-world functioning, patients also completed the Specific Levels of Functioning Scale (SLOF)^25^.

### Experimental design and procedures

#### Action stimuli

Stimuli were selected from a dataset of 900 reach-to-grasp movements obtained by recording 15 human naïve agents reaching, grasping, lifting, and moving a hazelnut or a grapefruit. For each agent and each object size, we selected the two representative reaching acts. The final set of stimuli consisted of 60 reaching acts (2 reaching acts × 15 agents × 2 object sizes). Detailed procedures and apparatus are described in ref. ^26^ and briefly summarized in Supplementary Material.

#### Procedure

Participants were seated in front of a 15.6-inch computer monitor (refresh rate 60 Hz, response time 8 ms) at a viewing distance of 50 cm. The task structure conformed to a two-alternative forced-choice task (Fig. 1B). In each trial, participants saw two videos, presented in random order, and separated by a 500 ms interstimulus interval. One video showed a hand reaching for a hazelnut (small object), the other video a hand reaching for a grapefruit (large object). To define the timecourse of information integration, reaching acts in each trial were presented under one of eight levels of temporal occlusion, from 10% up to 80% of movement duration. After viewing both videos, participants were prompted to indicate which video (first or second) contained the small (large) object by pressing with the index fingers one of two keys on a keyboard: a left key (i.e., “A”) when the interval containing the small (large) object was presented as the first interval, and a right key (i.e., “L”) when it was presented as the second interval. The prompt screen was displayed until response or for a maximum of 4000 ms. After response, participants were asked to rate the confidence of their choice on a four-level scale (from 1 = least confident, to 4 = most confident).

The experiment consisted of 240 trials split in eight blocks of 30 trials each. Participants were instructed to indicate the interval containing the small (large) objects in the first four blocks and the interval containing the large (small) objects in the last four blocks, counterbalanced across participants. Levels of occlusion were pseudorandomized and balanced across blocks so that each block contained at least one presentation of each of the eight levels of occlusion. At the end of each block, participants were informed about their mean accuracy over the 30 trials. To familiarize participants with the task, we administered ten practice trials. Stimuli, timing, and randomization procedures were controlled using a PsychToolbox script running in MATLAB R2014a (MathWorks, Inc.).

### Quantification and statistical analysis

The quantification and statistical analysis are detailed in Supplementary Material. In summary, we utilized Mixed Effects Models to examine the significance of the observer group (patient, control) and occlusion level (from 10% to 80% of movement duration) on the probability of choice (first vs. second) and the calibration of confidence-accuracy. To measure how size information encoded in movement kinematics was read by patients with psychosis and control observers at various occlusion levels, we employed the recently developed kinematic coding framework^16–18^. This framework enabled us to assess the encoding and readout of size information with single-trial resolution.

## Results

Participants completed a two-alternative object size discrimination task under eight levels of temporal occlusion, from 10% to 80% of movement duration. Each trial displayed two videos: one video displayed a reaching act towards a small object and the other video a reaching act towards a large object. The task was to indicate which video (first or second) contained the small (large) object. Full statistics of all comparisons are reported in Supplementary Tables 1–5. In all figures, * indicates *p* < 0.05, ** indicates *p* < 0.01, *** indicates *p* < 0.001.

**Fig. 2:**
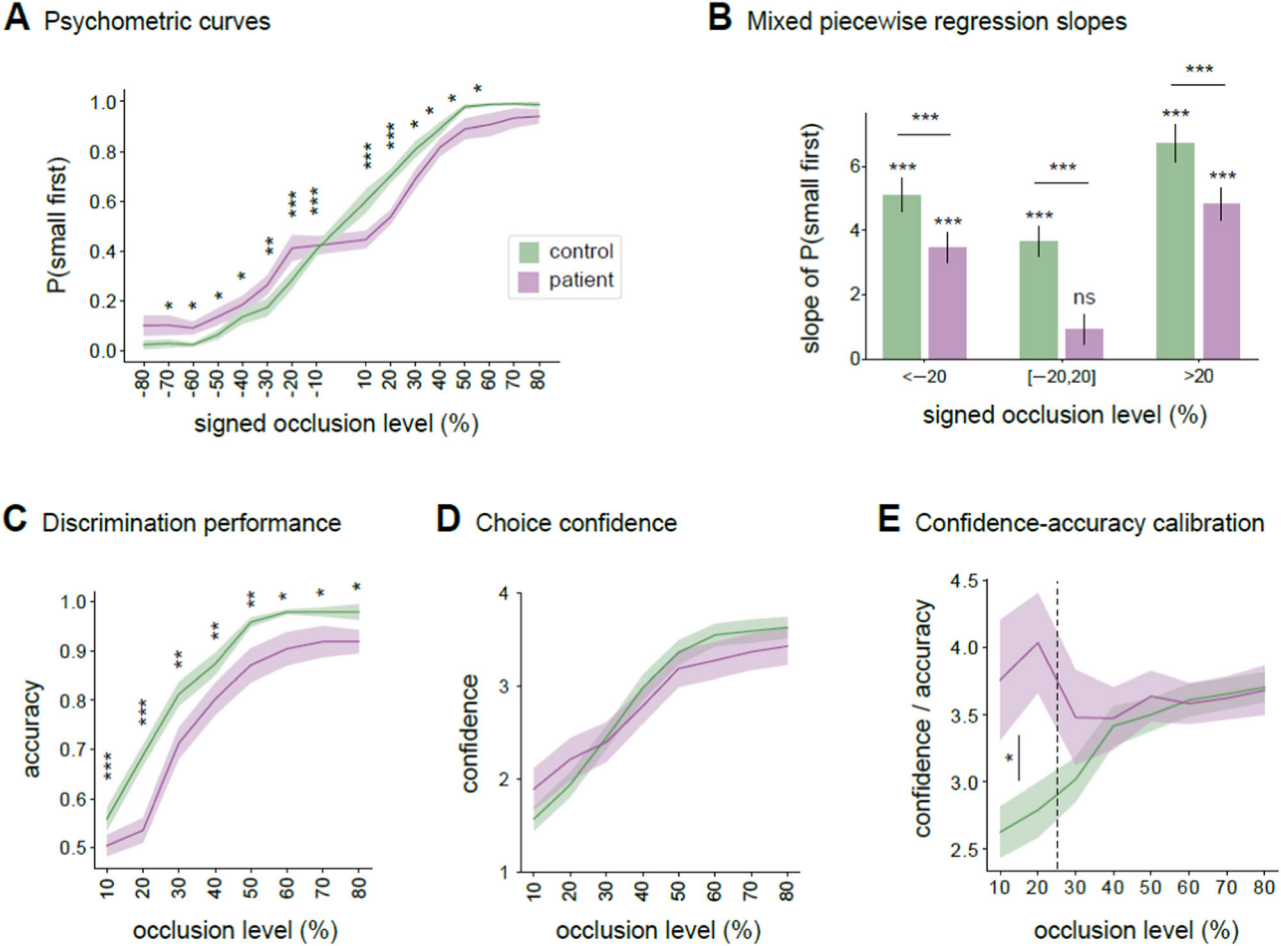
Results of action prediction. **A** Empirical psychometric curves quantifying the probability of responding ‘small first’, P(*small first*) as a function of occlusion level on ‘large first’ trials (represented as negative occlusion values) and ‘small first’ trials (represented as positive occlusion values) in observers with psychosis and controls. **B** Piecewise regression slopes of the psychometric curves as estimated by Logistic Mixed Effects Models with change points at ±20% in patients and control observers. Error bars represent standard errors. **C** Trial-averaged action prediction performance (fraction of correct responses) as a function of occlusion level in patients and control observers. **D** Reported confidence ratings as a function of occlusion level in patients and control observers. **E** Trial-averaged ratio between confidence ratings and action prediction accuracy as a function of occlusion level in patients and control observers. In (**A**), (**C**–**E**), solid lines and shaded areas represent mean ± SEM across participants. In (**A**), (**C**), and (**E**), asterisks denote significant differences between observer groups.

Psychometric curves quantifying the probability of responding ‘small first’ on ‘large first’ trials (represented as negative occlusion values) and ‘small first’ trials (represented as positive occlusion values) in observers with psychosis and controls are shown in Fig. 2A. Analyses using Mixed Effects Models revealed a significant interaction between observer group (patient, control) and occlusion level (from 10 to 80% of movement duration) (Supplementary Table 2), reflecting group differences in the rate of information integration across occlusion levels. As shown in Fig. 2A, while both groups exhibited an improvement in psychophysical performance with increasing occlusion level, patients displayed an overall reduced and discontinuous integration rate, with a pronounced central plateau (Fig. 2A). The fitting of a piecewise-sigmoidal psychometric function (see Supplementary Methods) identified a significant change point at ± 20%, marking the boundary of two integration periods: an initial period, from reach onset to 20% of movement duration, and a second period from 30% to 80% of movement duration (Supplementary Tables 2, 4). Patients exhibited lower performance than controls in both periods, but the difference between the groups was more than twice as large in the up-to-20% period (Fig. 2A). In this initial period, even though patients could discern size from specific movements (see Supplementary Figure 2), their psychometric curve slope (Fig. 2B; Supplementary Table 4) and overall prediction accuracy (Fig. 2C; Supplementary Table 4) were at chance level. These results suggest that patients with psychosis failed to integrate object size information in the period up to 20% of movement duration. Analyses including only patients diagnosed with schizophrenia or schizoaffective disorders showed qualitatively identical patterns of results (see Supplementary Fig. 1).

### Confidence-accuracy relationship across integration periods

The above results identify two distinct periods of information integration: 0–20% and 30–80% of movement duration. To assess whether the relationship between confidence ratings (Fig. 2D) and discrimination accuracy (Fig. 2C) differed between these periods, we calculated the ratio between confidence and accuracy. This ratio serves as a measure of confidence-accuracy calibration, quantifying how well variations in confidence track variations in accuracy (see Supplementary Methods). As illustrated in Fig. 2E, results using Mixed Effects Models revealed a significant interaction between observer group and integration period (Supplementary Table 2). In controls, the confidence/accuracy ratio followed the progression in accuracy, steadily increasing across the two integration periods. In patients, the ratio followed a trend similar to that of controls in the 30-80% period, but it diverged significantly in the initial 0–20% period. In this initial period, despite accuracy for patients markedly decreased compared to controls, their confidence-accuracy ratio was higher than that of the control group (Supplementary Table 4). This indicates that patients’ confidence did not track variations in accuracy in the initial integration period.

### Kinematic encoding and readout of object size information at the single-trial level

The results so far indicate an overall reduced pattern of integration in patients relative to controls, characterized by zero-integration period and a reduced calibration of confidence to accuracy up to 20% of movement duration. To determine the specific features patients extracted at different levels of temporal occlusion, whether these features differed from those extracted by the control group, and the effectiveness of information readout, we used the kinematic coding framework^16,17^. This framework was designed to quantify how intention information encoded in movement kinematics is read out by individual observers with single-trial resolution^16–18,27^. Here, we adapted it to measure how patients and controls read size information prospectively encoded in single-trial kinematics across levels of occlusion.

**Fig. 3:**
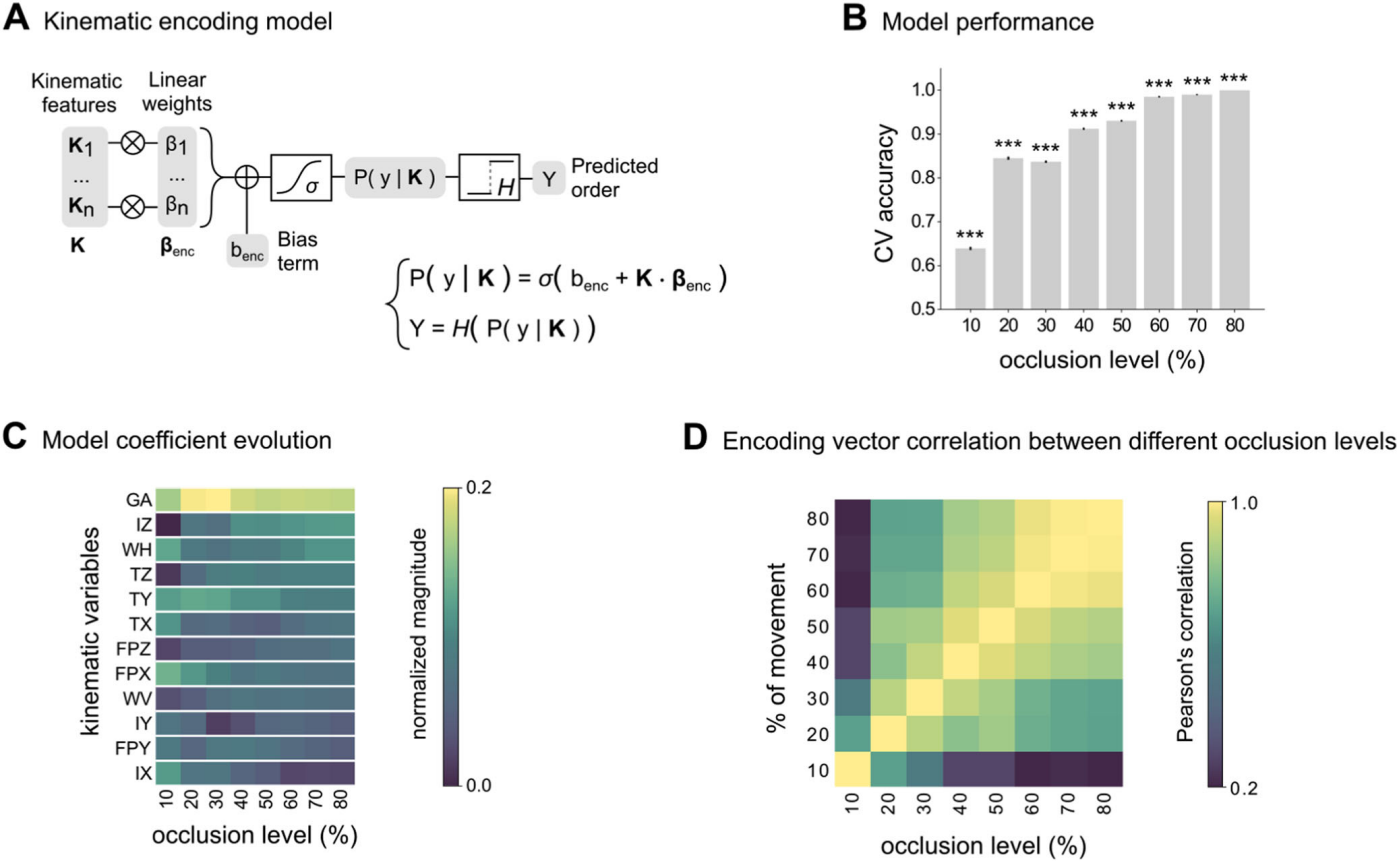
Encoding of size information from single-trial kinematics. **A** Block diagram and equation of the kinematic encoding model used to quantify size information at a given occlusion level. Β_enc_ is the model linear coefficient vector, b_enc_ is the intercept coefficient, *σ* is the sigmoid function, and *H* is the Heaviside function. **B** Cross-validated (CV) performance of kinematic encoding models as a function of occlusion level. Bars represent mean ± SEM across stimuli. **C** Contribution of individual kinematic features to kinematic encoding of size information. Kinematic variables (for acronyms, see Supplementary Methods) are ordered by decreasing coefficient magnitude in the encoding model fitted at the 80% occlusion level. **D** Pearson’s correlation of coefficient distribution across kinematic variables between different occlusion levels.

As a first step to assess the availability of size information in single-trial kinematics across varying levels of occlusion, we employed a set of encoding models. For each occlusion level, we computed an encoding model on the kinematics of the reaching acts shown across trials. The encoding model predicts the probability of the small object being reached within the first interval in each trial based on the differences between the kinematics of two reaching actions shown within that trial (Fig. 3A; see Supplementary Material). We quantified size information at each level of occlusion as the cross-validated accuracy of the encoding model in predicting the size of the object to be grasped.

As shown in Fig. 3B, size information was already encoded at 10% of movement duration, exhibited more than twofold increase at 20%, and continued to steadily rise, surpassing 95% at 60% of movement duration (Fig. 3B; Supplementary Tables 3 and 5). Figure 3C visualizes the contribution of individual kinematic features to size encoding as measured by the normalized magnitude of regression coefficients of the feature in the encoding model at each occlusion level. Consistent with^26^, the most informative feature, grip aperture (GA), encoded size information as early as 10% of movement duration, with its contribution peaking at 20 and 30% of movement duration. In other variables, such as index finger height (IZ), size encoding increased as time progressed. Finally, other variables such as wrist height encoded size more stably across time. Overall, the pattern of encoding showed a high stability from 40% of movement duration as quantified by the correlation of the encoding vectors at different occlusion levels (Fig. 3D).

**Fig. 4:**
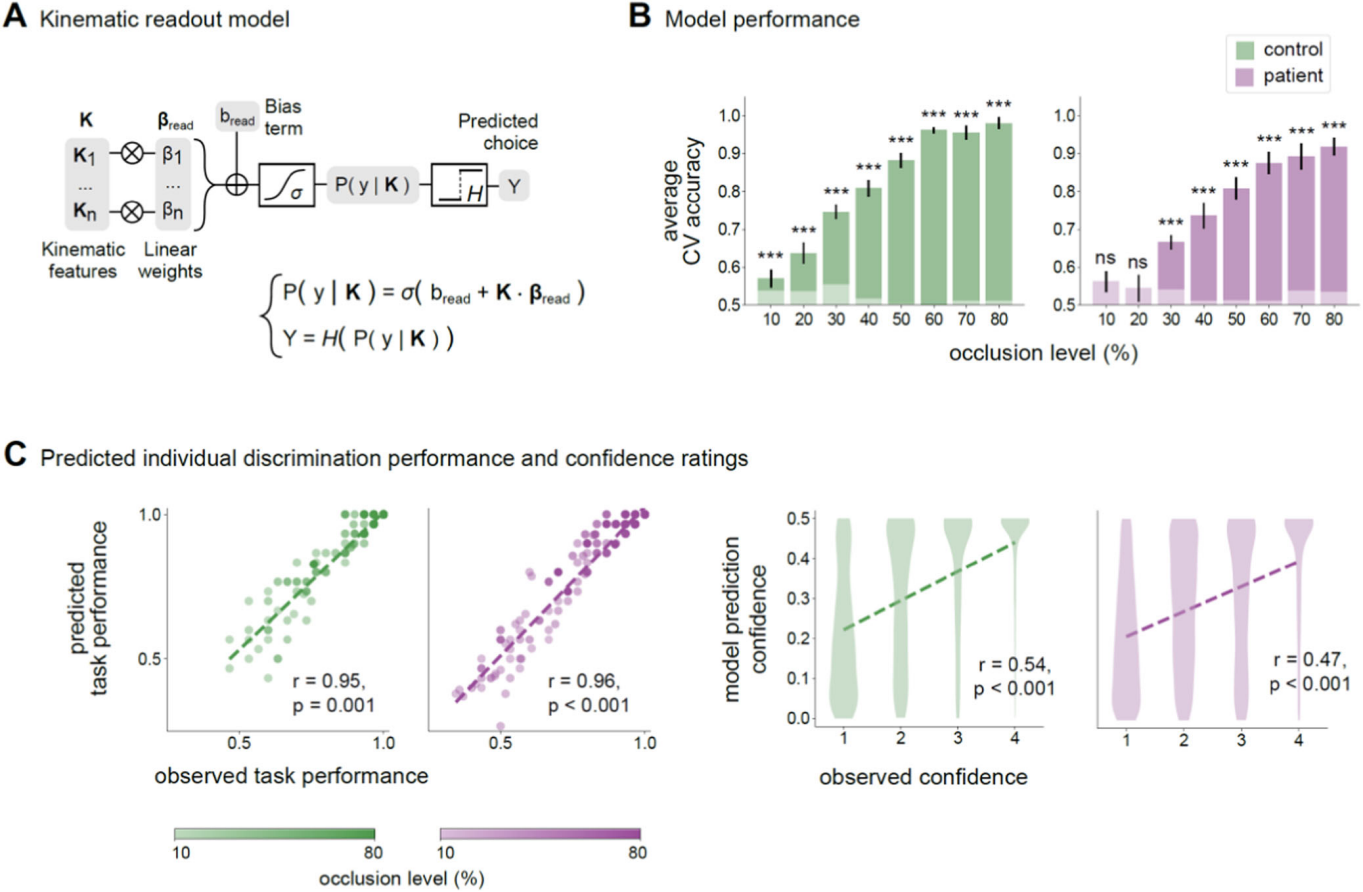
Readout of size information from single-trial kinematics. **A** Block diagram and equation of the individual-participant kinematic readout model used to quantify the readout of size information from single-trial kinematics at a given occlusion level. β_read_ is the model linear coefficient vector, b_read_ is the intercept coefficient, *σ* is the sigmoid function, and *H* is the Heaviside function. **B** Cross-validated (CV) performance of kinematic readout models as a function of occlusion level for control observers and observers with psychosis. Bars represent mean ± SEM across participants. The light sub-bars represent chance-level performance, quantified as the mean of the null-hypothesis distribution of cross-validated model performance. **C** Left panels. Scatter plots of the relationship between the observed size discrimination performance and the performance predicted by the kinematic readout model across individual participants in the control group and in the patient group. Darker shades correspond to later occlusion levels. Right panels. Violin plots of the within-group relationship between reported confidence ratings and model prediction confidence rating, computed as the deviation of the estimated probability of answering “small first” from chance. Fitted regression lines are displayed over the data. Pearson’s correlation coefficient (r) and their significance values (p) are reported.

The encoding model quantifies the size information encoded in movement kinematics and potentially available to an ideal observer^18^. To determine how well observers in each group read such information, we trained a set of readout models (Fig. 4A). The readout model computes, separately for each observer and each occlusion level, the probability of reporting the small object being reached within the first interval based on the disparity between the kinematics of two reaching acts within that trial. The term $\vec{K}\cdot \vec{\beta }$ describes how the individual observer integrates kinematic evidence, while the term ${\beta }_{0}$ describes the bias towards choosing ‘small first’ versus ‘large first’ independent of the evidence. In both groups, the fractional contribution of evidence-independent bias to readout was very small (<0.01) and not significant across all occlusion levels (*p* > 0.15 for controls and *p* > 0.63 for patients). This indicates that variations in readout were due to how individual observers integrated kinematic evidence.

The tight correlation between predicted and observed individual accuracies indicates that the readout model accurately captured the dependency of observers’ choice on single-trial kinematics at the single-observer level (Fig. 4C). As shown in Fig. 4C, although confidence ratings were not used for model fitting, the model also accurately predicted the confidence with which observers endorsed single-trial size choices. These results indicate that our readout model was able to predict how well and how confidently individual observers predicted object size from single-trial kinematics.

As shown in Fig. 4B, for both groups, readout model performance increased across progressive occlusion levels (Supplementary Table 3). Only for patients, readout model performance was at chance at 10% and 20% of movement duration. This indicates that in this initial period, patients’ choices did not depend on single-trial movement kinematics (Fig. 4B; Supplementary Table 5). Supporting these findings, an analysis of the information read at the single-trial level revealed the patients extracted barely any of the encoded information in the initial period (Supplementary Fig. 3).

### Readout profiles of individual observers

To determine which kinematic features observers in each observer group read (and failed to read), we next computed the contribution of each feature to the readout of object size information as the normalized regression coefficient (weight) of the feature in the readout model.

**Fig. 5:**
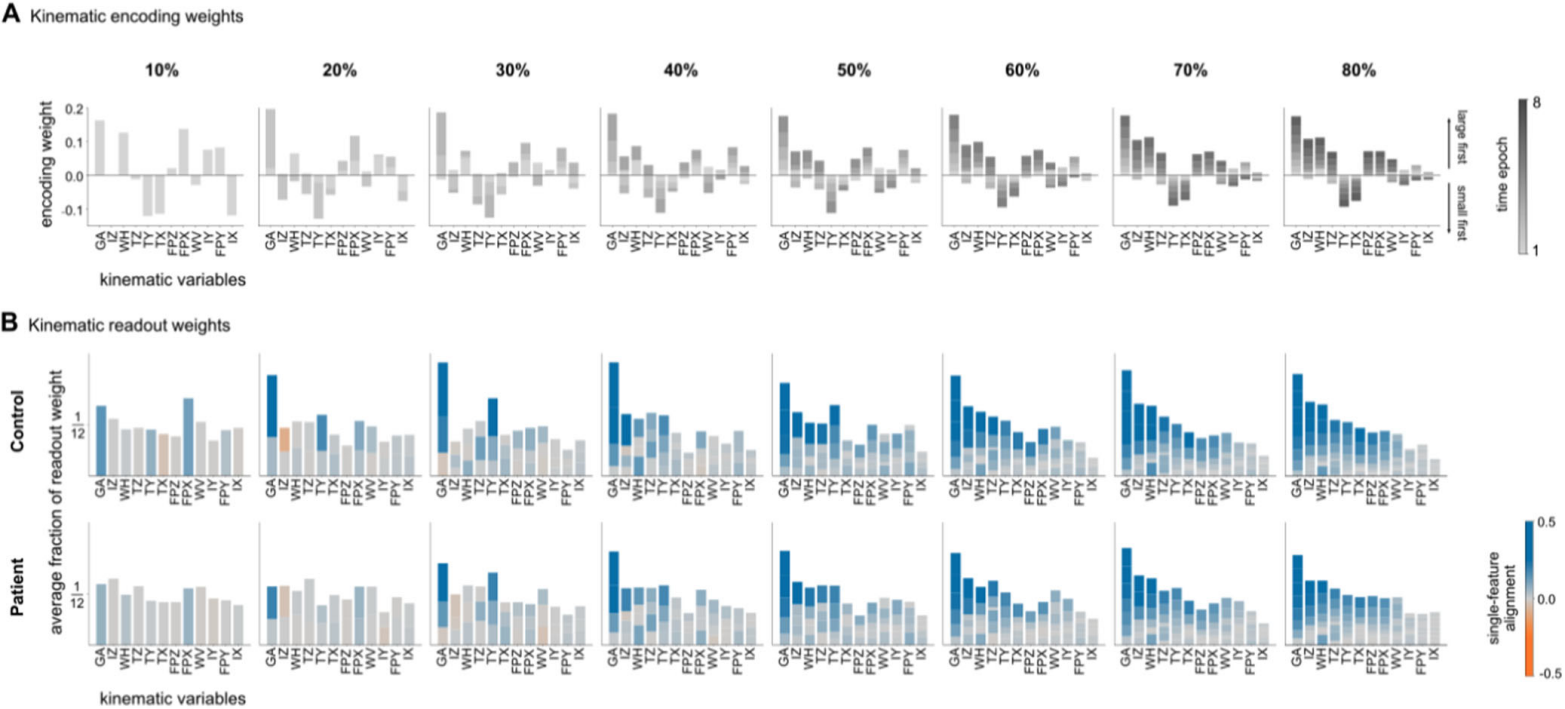
Distribution of kinematic readout weights relative to encoding weights. **A** Encoding model weights normalized by the total encoding weight at each occlusion level. Different shades of stacked bars indicate time epochs. Across occlusion levels, kinematic variables are ordered by the value of the encoding weight at the 80% occlusion level (in descending order). **B** Average fraction of readout weights across participants at each occlusion level in the control group and in the patients group. Different colors of stacked bars indicate single-feature alignment of readout weights relative to encoding weights. Stacking order reflects the order of time epochs (from bottom to top). The ordering of kinematic variables (for acronyms, see Supplementary Methods) is the same as in (**A**).

The alignment of readout weights (Fig. 5B) relative to encoding weights (Fig. 5A) provides an intuitive measure of how well observers in each group read the information encoded in each feature. The prevalence of positively aligned readout weights (denoted by blue bars in Fig. 5B) indicates that observers in both groups generally extract the encoded information accurately. However, relative to controls, observers with psychosis read little if any size information from individual features at 10% and 20% of movement duration. This is evident when looking at GA. Observers in the control group correctly read size information encoded in this feature as early as 10% and 20% of movement duration. Observers with psychosis read little information in GA (and any other feature) up to 30% of movement duration.

### Relation to illness, antipsychotic medication, and symptoms

To explore the relation of action prediction to illness and medication, we examined the correlation between individual action prediction performance and individual readout strength with illness duration, illness onset, and antipsychotic medication as measured by chlorpromazine equivalents. As shown in Supplementary Fig. 3, these analyses found no significant correlations. Additionally, we examined the correlation of action prediction performance and individual readout strength with symptoms as measured by BPRS, BNSS, PANSS, PANNSp, PANSSn, SLOF (social), SLOF (relationship), SLOF (acceptability), NES, and TMT(B-A) (Supplementary Fig. 4). The results indicated no significant correlation of with positive symptoms or cognitive functioning. However, there was a significant negative correlation between BNSS and action prediction accuracy and BNSS and individual readout strength. This indicates that patients with more severe negative symptoms had greater difficulties in predicting actions and reading size information. The only other significant correlation was between SLOF functioning in social relationships and prediction accuracy; patients less able to predict others’ actions also showing reduced functioning in relationships.

## Discussion

The ability to anticipate what others will do next is crucial for navigating social, interactive environments (Becchio et al.^28^). Imagine sitting at a dinner table with a friend. Is she about to reach for her empty glass or the bottle? If she is aiming for the glass, you should prepare to pour some wine; if for the bottle, you should be ready to hold out your glass instead. Movement kinematics provides rapid access to this information^15,18^. As soon as 80 ms after the onset of the action, healthy observers are able to anticipate the action’s outcome. This rapid perception enables them to quickly formulate and carry out an appropriate response to the unfolding action. Here, we provide evidence that this predictive ability is impaired in patients with psychosis. By combining a temporal occlusion paradigm with our kinematic coding framework, we were able to rigorously quantify the information encoded and readout across levels of occlusion (from 10% to 80% of movement duration) and demonstrate an overall pattern of reduced and discontinuous evidence integration in patients with psychotic disorders.

Patients exhibited a period of null integration (represented as a central plateau) during the initial phase of the action, up-to-20% of movement duration. In psychometric distributions, plateaus in evidence integration are rarely observed or reported, possibly due to the fact that temporal intervals are often too broadly spaced for plateaus to be visible^29^. Our approach enabled us to reveal a discontinuity in the psychotic integration pattern, which would have remained unnoticed with fewer temporal intervals or by solely examining later intervals^14^. Our single-trial analysis allowed us to further demonstrate that this initial plateau reflected an inability to integrate object size information encoded in single-trial kinematics. These results add to the existing evidence of impaired processing of human motion in psychosis^30^ by showing a specific impairment in extracting information during the initial stages of an action.

Early access to information encoded in movement kinematics has been linked to interpersonal motor resonance^31,32^. The finding of a lack of attunement to advanced information in movement kinematics among individuals with psychosis could reflect abnormalities in the time course of interpersonal motor resonance. Partial support for this hypothesis comes from studies documenting abnormalities in early motor resonance but not late motor resonance in patients with schizophrenia when observing action sequences^33^. However, this work primarily examined interpersonal motor resonance on a scale of seconds to minutes (5–200 s). It remains to be determined whether alterations in the time course of interpersonal motor resonance also occur on the faster scale (i.e., 10–100 of milliseconds) relevant for action prediction.

Contrary to what one might expect in typical observers^29^, in patients, reduction of accuracy in the up-to-20% integration period relative to controls was not accompanied by a reduction in confidence. Individuals with psychosis generally exhibit a tendency to “jump to conclusions”, a cognitive bias that has been linked to proneness to unstable belief formation^34^, and is especially prominent during the acute phases of psychotic disorders^35^. We speculate that patients’ reduced calibration of confidence to accuracy during the up-to-20% integration period may share a common mechanism with, and potentially contribute to, this bias. Combined with underweighting of prior beliefs^36^, reduced calibration could lead to reduced data-gathering, premature decisions, and endorsement of odd beliefs in their daily lives^37^. To explore this hypothesis, future studies could probe the influence of priors on the integration of information in movement kinematics (for an example of prior manipulation, see ref. ^38^) and its relationship to delusion proneness.

In our study, we found no relationship between difficulties in action prediction and the severity of positive symptoms. However, we observed a relationship between prediction difficulties and both the severity of negative symptoms and the decline in social relationship functioning. Patients less able to predict others’ actions also exhibited more severe negative symptoms and reduced functioning in social relationships. These findings, together with existing evidence of under-weighing and under-counting of information from others^39^, are of clinical relevance since they suggest that patients with psychosis may lose interest in social interactions partly due to their difficulties in anticipating and responding to the actions of others. Our results do not establish a causal relationship between action prediction abnormalities, negative symptoms, and social functioning. However, if replicated in a larger sample and found to be causal, action prediction deficits may serve as a promising target for improving social cognition in patients with psychosis.

It is a long-standing notion in phenomenological psychiatry that patients with psychosis disorders are characterized by disturbances of *intercorporeality*, the pre-reflective intertwining of living bodies^40^. Our findings support and refine this notion by revealing how abnormal patterns of information readout prevent rapid, implicit access to others’ goals.

### Supplementary information

Supplemental Material
