# Supplementary material for: Action prediction in psychosis
Source: Schizophrenia (Heidelb). 2024 Jan 10;10(1):8. doi: 10.1038/s41537-023-00429-x (PMC10851700; doi:10.1038/s41537-023-00429-x)
Supplement: Supplementary file 1 — Supplemental Material [file 41537_2023_429_MOESM1_ESM.docx]

**Supplementary Information for**

Action prediction in psychosis

**This file includes:**

Supplementary Methods

Supplementary Figures 1 to 5

Supplementary Tables 1 to 5

**Supplementary Methods**

**Sample size**

We determined the sample size prior to data collection with a power analysis (G*Power V 3.1.9.7) based on the estimate of the smallest expected effect size, which is the deviation of accuracy from chance at 10% of movement duration. For this, we had a precise estimate from a previous study of our group in healthy volunteers (Ansuini et al., 2016). The parameters for the power analysis were as follows:

Mean H0 = 0.5

Mean H1 = 0.57 (accuracy at 10% of movement duration)

SD = 0.074

Effect size (Cohen's D) = 0.946

The result of the power analysis is that a minimum of 12 participants is needed to achieve 90% power to detect the smallest effect (α = 0.05; one-tailed) in each group. Of course, detecting other effects requires less samples. For example, detecting accuracy above chance at 20% of movement duration requires 6 participants.

Expecting some attrition, we recruited 16 participants for each group.

**Supplementary details for action stimuli**

Stimuli were selected from a dataset of 900 reach-to-grasp movements obtained by recording 15 human agents reaching, grasping, lifting, and moving a hazelnut (diameter = ~1.5 cm; weight = ~2 g) or a grapefruit (diameter = ~10 cm; weight = ~354 g). Each agent performed a total of 60 action sequences (30 actions for each object size). Detailed procedures and apparatus are described in (Ansuini et al., 2015). In brief, actions were tracked using a near-infrared camera 3D motion capture system (frame rate, 100 Hz; Vicon System) and simultaneously filmed from a lateral viewpoint using a digital video camera (Canon Alegria, 25 frames/s). The agent’s right hand was outfitted with 11 retroreflective hemispheric markers to compute the following kinematic variables of interest:

- wrist velocity (WV), defined as the module of the three-dimensional velocity vector of the wrist marker (in mm/s);
- wrist height (WH), defined as the z-component of the wrist marker (in mm);
- grip aperture (GA), defined as the Euclidean distance between the tip of the thumb and the tip of the index finger;
- x-index (IX), y-index (IY), and z-index (IZ), defined as the x-, y-, and z-coordinates of the tip of the index finger (in mm);
- x-thumb (TX), y-thumb (TY), and z-thumb (TZ), defined as the x-, y-, and z-coordinates of the tip of the thumb (in mm);
- x-dorsum plane (DPX), y-dorsum plane (DPY), and z-dorsum plane (DPZ), defined as the x-, y-, and z-components of the radius-phalanx plane. This plane provides information about the abduction, adduction, and rotation of the hand dorsum independent of the rotation of the wrist.

Custom software written in MATLAB (MathWorks Inc.) was used to extract the selected variables. Each variable was calculated at intervals of 10% of the movement duration from reach onset to reach offset. For each agent and each object size, we selected the two representative reaching acts (i.e., to acts that minimized the Euclidean distance with the average kinematics computed across all agents and trials). The final set of stimuli consisted of 60 reaching acts (2 reaching acts x 15 agents x 2 object sizes). Digital video editing (Adobe Premiere Pro; .avi format, disabled audio, 25 frames/s) was used to occlude the to-be-grasped object. Each movie started at reach onset and ended at reach offset. Actions were tracked using a near-infrared camera 3D motion capture system (frame rate, 100 Hz; Vicon System) and simultaneously filmed from a lateral viewpoint using a digital video camera (Canon Alegria, 25 frames/s).

**Quantification and statistical analysis**

***Psychometric curves.*** We re-coded participants’ choices in the 2AFC task as ‘small first’ and ‘large first’ choices, and computed, separately for each group, the psychometric curve of the probability of choosing ‘small first’ as a function of the level of occlusion. In Figure 2A, positive and negative occlusion values in the x-axis indicate ‘small first’ and ‘large first’ trials, respectively. We used Logistic Mixed Effects Models to assess the significance of the effect of temporal occlusion on the choice probability *P*(small first) between observer groups. This allowed us to fit population-level psychometric curves while controlling for inter-subject variability (Moscatelli, Mezzetti, & Lacquaniti, 2012). We considered choice (first or second interval) as dependent variable, observer group, occlusion level, and their interaction as fixed effects, age and education as covariates, and subject (random intercept, and random slope of occlusion level), block (random intercept), and block randomization (large/small, random intercept) as random effects. This resulted in the following full-model equation:

$$choice \sim\sigma\left( w_{1} group + w_{2} occlusion + w_{3} group*occlusion+w_{4} age+w_{5} education+ w_{6}^{i} occlusion + w_{7}^{i} + w_{8}^{j}+w_{9}^{k} \right)$$

where $\sigma$ is a sigmoidal function mapping the values in the (0,1) interval to model accuracy saturation; $w_{1}, w_{2} , w_{3}, w_{4}, w_{5}$ are the fixed-effect weights*;* $w_{6}^{i}, w_{7}^{i}, w_{8}^{j}, w_{9}^{k}$ *(*$i=1, \ldots, N_{subjects}, j=1, \ldots, N_{blocks}, k=large, small$*)* are the random effect weights (Supplementary Table 2). Selection of fixed and random effects was performed as detailed in “Selection of random and fixed effects structure in Mixed Effects Models” (see below).

We fitted piecewise versions of the model detailed above, to allow for varying slopes of the psychometric curves at different occlusion levels (as suggested by Figure 2A). Specifically, we included symmetrical change points at ±10%, ±20%, or ±30%. We compared the three piecewise models with a non-piecewise model and verified that the piecewise model with symmetrical change points at ± 20% yielded the best performance as measured by the Bayesian Information Criterion (BIC) (Schwarz, 1978) (Supplementary Table 2). We thus defined the following integration periods based on the identified change points: < -20%, [-20,20], > 20%. Within each period, we used the linear coefficients within the piecewise model to quantify the slope of the psychometric curve (Figure 2B). The slope quantifies the rate of change of the psychometric function, that is, the rate at which the probability of a correct response changes within each integration period as a function of the occlusion level.

***Accuracy****.* We conducted a similar analysis on accuracy by pooling together ‘small first’ and ‘large first’ trials and considering single-trial accuracy as dependent variable. To assess potential learning effects and their variation between groups, our analysis also incorporated block and its interaction with observer group as a fixed effect. This resulted in the following full-model equation:

$$accuracy \sim\sigma\left( w_{1} group + w_{2} occlusion + w_{3} group*occlusion+w_{4} block+ w_{5} group*block+w_{6} age+w_{7} education+ w_{8}^{i} occlusion + w_{9}^{i} \right)$$

where $\sigma$ is a sigmoidal function mapping the values in the (0,1) interval to model accuracy saturation; $w_{1}, w_{2} , w_{3}, w_{4}, w_{5}, w_{6}, w_{7}$ are the fixed-effect weights*;* $w_{8}^{i}, w_{9}^{i}$ *(*$i=1, \ldots, N_{subjects}$*)* are the random effect weights (Supplementary Table 2).

We found a significant effect of block on accuracy (see Supplementary Table 2). However, the interaction between block and observer group did not reach statistical significance. This indicates that changes in prediction accuracy over time were comparable between the groups.

***Confidence-******accuracy calibration.*** To measure the relationship between confidence and accuracy at different occlusion levels, we computed the ratio between the confidence ratings and the prediction accuracy of a given observer at a given occlusion level. This ratio serves as a measure of confidence-accuracy calibration, quantifying how well variations in confidence track variations in accuracy. An observer who discriminates object size with perfect accuracy (accuracy = 1) and responds with the highest confidence (confidence = 4) would have a confidence/accuracy ratio of 4. An observer who discriminates object size with chance accuracy (accuracy = 0.5) and responds with the lowest confidence (confidence = 1) would have a confidence/accuracy ratio of 2. Therefore, if an observer's confidence tracks their accuracy, we would expect this ratio to increase from 2 to 4 as accuracy improves from 0.5 to 1.

We created a calibration curve by plotting confidence-accuracy calibration values as a function of occlusion level (Figure 2E). We used Gamma Mixed Effects Models to assess the significance of the difference in confidence-accuracy calibration between groups over the two integration periods. We chose a Gamma distribution because the confidence-accuracy data were non-negative and positively skewed (Ng & Cribbie, 2016). We compared gamma distributions with other distributions which allow for skewness (inverse gaussian, lognormal) and verified that models with gamma distributions performed better in terms of log-likelihood. We considered confidence-accuracy calibration as dependent variable, group and integration period, as well as their interaction, as fixed effects, age and education as covariates, and subject (random intercept, and random slope of integration period) and session block (random intercept) as random effects. This resulted in the following full-model equation:

$$calibration \sim h\left( w_{1} group + w_{2} \left( integration period \right)+ w_{3} group*\left( integration period \right)+w_{4} age+w_{5} education+ w_{6}^{i} \left( integration period \right)+ w_{7}^{i} + w_{8}^{j} \right)$$

where $h$ is the inverse link function; $w_{1}, w_{2} , w_{3}, w_{4}, w_{5}$ are the fixed-effect weights; and $w_{6}^{i}, w_{7}^{i}, w_{8}^{j}$ *(*$i=1, \ldots, N_{subjects}, j=1, \ldots, N_{blocks}$*)* are the random effect weights (Supplementary Table 2).

***Kinematic encoding and readout of size information.*** To model single-trial kinematics, we averaged the 12 kinematic variables of interest over 10 time epochs of 10% of the normalized movement duration. For each occlusion level (from 10% to 80% of movement duration), we created an n-dimensional vector, with dimensions ranging from 12 features (12 kinematic variables over 1 time epoch) for the 10% occlusion level to 96 features (12 kinematic variables over 8 time epochs) for the 80% occlusion level. Next, based on (Patri et al., 2020), we computed the difference between the kinematics of two reaching acts in each trial as:

$$\vec{K} = \vec{K_{1}} - \vec{K_{2}}$$

where $\vec{K_{1}}$ and $\vec{K_{2}}$ are the kinematic vectors associated with reaching acts displayed in the first and second interval. This definition reflects the assumption that, in a 2AFC task, choices are based on comparative judgements. To quantify the kinematic encoding of size information, for each occlusion level, we trained a logistic regression model to predict the single-trial probability that the small object was presented in the first interval $Y$ as a sigmoidal function of a linear combination of the components of the single-trial kinematic vector $\vec{K}$. The equation was as follows:

$$P\left( Y=‘small first’ | \vec{K} \right)=\sigma\left( \vec{K}\cdot\vec{\beta}+ \beta_{0} \right);$$

$$P\left( Y=‘large first’ | \vec{K} \right)=1-P\left( Y=‘small first’ | \vec{K} \right)$$

where $\sigma$ is the sigmoid function, $\vec{\beta}$ is the vector containing the values of the regression coefficients of each kinematic feature, and $\beta_{0}$ is the kinematic-independent bias term. The length of the weight vector $\vec{\beta}$matched the dimension of the kinematic vector $\vec{K}$ for the considered temporal occlusion. Logistic regression was implemented using the *pyglmnet* Python library (Jas et al., 2020).

We used a similar set of logistic regression models to analyze how size information was read out by individual observers in each group (kinematic readout of size information). For each participant and each occlusion level, we trained a logistic regression model to predict the single-trial probability of the observer reporting the small object to be reached within the first interval *Y* as a sigmoidal function of a linear combination of the components of the single-trial kinematic vector $\vec{K}$. Kinematic readout models were defined as in the above equation, but with the binary stochastic variable $Y$ representing the interval chosen by the participant (Figure 3A). The term $\vec{K}\cdot\vec{\beta}$ describes how the individual observer integrates kinematic evidence, while the term $\beta_{0}$ describes the bias towards choosing ‘small first’ versus ‘large first’ independent of evidence. To assess the relative contribution of the evidence-independent bias to action prediction, we computed the fractional contribution of the bias to readout (as the ratio ${|\beta}_{0}|/({|\beta}_{0}|+|\vec{K}\cdot\vec{\beta}|)$ and tested it against the null-hypothesis distributions obtained by fitting the readout model on permuted data.

***Training and evaluation of logistic regression models.*** Training and evaluation were performed similarly for encoding and readout models. All models were trained separately for each level of occlusion and, in the case of readout models, individually for each participant. To avoid penalizing predictors with larger ranges of values, we z-scored single-trial kinematic vectors within each model. Models were trained using L^2^ regularization. The parameter $\lambda$, which controls the strength of the regularization term, was estimated for each model using leave-one-out cross-validation. We retained for each model the value $\lambda_{min}$ associated with the minimum mean cross-validated error. Models were fitted on the whole training set with the retained regularization term.

We evaluated the performance of the encoding and readout models by repeated 5-fold cross-validation (50 random splits) (Kim, 2009), on top of the cross-validation used for the determination of the $\lambda$ parameter. We computed the most likely value of *Y* for each trial by taking the argmax over *Y* of $P\left( \vec{K} \right)$. Model performance was quantified as the fraction of correct predictions averaged over folds and random splits. For each level of occlusion, we created a chance-level null-hypothesis distribution of model performance by fitting the models after randomly permuting across trials the binary variable $Y$.

We used Linear Mixed Effects Models to quantify the effect of occlusion level and group on encoding and readout model performance, and to compare model performance against chance and between groups. We used the fraction of correct model predictions of each video across cross-validation repetitions as dependent variable. For encoding model performance, we considered occlusion level as fixed effect, and video ID (random intercept) as random effect, resulting in the following full-model equation:

$$fraction correct \sim w_{1} occlusion + w_{2}^{k}$$

where $w_{1}$ is the fixed-effect weight; $w_{2}^{k}$ *(*$k=1, \ldots, N_{videos}$) are the random effect weights (Supplementary Table 3).

For readout model performance, we considered occlusion level, group and their interaction as fixed effects, and subject (random intercept, and random slope of occlusion level) and session block (random intercept) as random effects, resulting in the following full-model equation:

$$fraction correct \sim w_{1} group + w_{2} occlusion + w_{3} group*occlusion + w_{4}^{i} occlusion + w_{5}^{i} + w_{6}^{j}$$

where $w_{1}, w_{2} , w_{3}$ are the fixed-effect weights; $w_{4}^{i}, w_{5}^{i}, w_{6}^{j}$ *(*$i=1, \ldots, N_{subjects}, j=1, \ldots, N_{blocks}$*)* are the random effect weights (Supplementary Table 3).

Although there was no overall response bias, the ratio between ‘small first’ and ‘large first’ responses varied across observers and occlusion levels – thus determining different chance-level null-hypothesis distributions of readout model performance. We estimated null-hypothesis distributions, separately for each participant and each occlusion level, by fitting the model on permuted data. To make all readout model performance values comparable, we then z-scored all values for a given participant and occlusion level using the mean and standard deviation of the single-subject, single-occlusion null-hypothesis distribution. Having verified that the coefficient vectors obtained by averaging the model weights over cross-validation folds showed near-perfect Pearson’s correlation with the coefficient vectors obtained by re-training the models on all trials, we used the latter for all post-hoc analyses of regression coefficient vectors.

***Action prediction performance and confidence predicted by the readout model.*** In Figure 4C, we used kinematic readout models to estimate the action prediction performance and confidence ratings of individual participants in each group. Using Eq. 1, we computed the interval choice predicted as most likely by the readout model for each trial and compared it to the actual order of the presented stimuli. Predicted action prediction performance was obtained by averaging the probability of correct interval choice across all trials for a given participant. The resulting value was then compared to the observed discrimination accuracy of the participant. By the same logic, we computed the confidence of single-trial model predictions as deviations of the estimated probability of reporting ‘small first’ from chance (0.5) and compared them with the confidence ratings reported by participants (Figure 4C).

***Contribution of individual kinematic features to encoding and readout.*** We computed the contribution of each kinematic feature to kinematic encoding (readout) as the feature regression coefficient in the encoding (readout) logistic regression model. A positive (negative) sign is assigned to a feature distributed across trials with higher (lower) values for ‘small first’ compared to ‘large first’.

***Single-feature alignment between encoding and readout.*** We quantified, separately for each observer and each level of occlusion, the alignment of readout coefficients relative to encoding coefficients at the single feature level. This was computed as the product between the encoding and readout weights of that feature, weighted by the norm of the whole encoding and readout vectors and adjusted by number of time epochs to make its values comparable across different occlusion levels:

$$alignment [i] \left( \vec{\beta}_{enc} , \vec{\beta}_{read} \right)=\frac{{\beta^{i}}_{enc} {\beta^{i}}_{read}}{\left| \left| \vec{\beta}_{enc} \right| \right| \left| \left| \vec{\beta}_{read} \right| \right|}\cdot t$$

where *t* ∈ (1,…,8) t is the number of time epochs; ${\beta^{i}}_{enc}$ and ${\beta^{i}}_{read}$ are the regression weights of the $i$-th kinematic feature in the encoding and readout model, respectively; $\vec{\beta}_{enc}$ and $\vec{\beta}_{read}$ are the whole encoding and readout coefficient vectors. High positive values (large encoding weights) indicate that a feature is highly informative and is correctly readout with large readout weights; high negative values indicate that a feature is highly informative and is incorrectly readout with large readout weights. Alignment values close to zero indicate that a feature is weakly informative, weakly read-out, or both.

***Selection of random and fixed effects structure in Mixed Effects Models.*** As in (Montobbio et al., 2022), we applied a backward model selection procedure, starting from the model with the most complex structure to arrive at a model that included only the significant predictors. We first selected the random effect structure of the model by keeping the full fixed effect structure and using the BIC. The BIC rewards model fit and penalizes model complexity. We then retained the optimal random effect structure and selected the best fixed effect structure by conducting likelihood-ratio tests (LRT) between models differing only by the presence or absence of one predictor (Agresti, 2007). Model selection results are reported in Supplementary Tables 2 and 3. We performed model fitting using the R package *lme4* (https://CRAN.R-project.org/package=lme4). We performed comparisons against chance and across levels of the selected models using the R package *emmeans* (https://CRAN.R-project.org/package=emmeans). The *emmeans* package estimates the marginal means and standard errors over combinations of predictors, from which *z*-values (to calculate two-sided *p*-values) are computed. Statistical comparisons for the effects are reported in Supplementary Tables 4 and 5.

***Conventions for p-values.*** Supplementary Tables 2-5 report details of Generalized Mixed Effects Models statistical tests and non-parametric permutation tests. Reported *p*-values are two-sided and Holm-Bonferroni corrected for the number of comparisons listed for each entry. In all figures, * indicates *p* < 0.05, ** indicates *p* < 0.01, *** indicates *p* < 0.001, *ns* indicates *p ≥* 0.05. Following standard notation, asterisk(s) above bars indicate significance of difference from chance of an individual quantity, asterisk(s) above brackets indicate significance of difference between two quantities.

***Statistical significance of correlations.*** The significance of Pearson’s correlation values in Figure 4C and Supplementary Figures 1-2 was assessed using the *stats* module from Python package *SciPy* (Virtanen et al., 2020) with two-sided parametric Student statistics.

**Supplementary references**

Agresti, A. (2007). *An Introduction to the Categorical Data Analysis*. John Wiley & Sons, Inc.

Ansuini, C., Cavallo, A., Koul, A., D'Ausilio, A., Taverna, L., & Becchio, C. (2016). Grasping others' movements: Rapid discrimination of object size from observed hand movements. *J Exp Psychol Hum Percept Perform*, *42*(7), 918-929. <https://doi.org/10.1037/xhp0000169>

Ansuini, C., Cavallo, A., Koul, A., Jacono, M., Yang, Y., & Becchio, C. (2015). Predicting object size from hand kinematics: a temporal perspective. *PloS One*, *10*(3), e0120432. <https://doi.org/10.1371/journal.pone.0120432>

Jas, M., Achakulvisut, T., Idrizović, A., Acuna, D., Antalek, M., Marques, V., Odland, T., Garg, R., Agrawal, M., Umegaki, Y., Foley, P., Fernandes, H., Harris, D., Li, B., Pieters, O., Otterson, S., De Toni, G., Rodgers, C., Dyer, E., . . . Ramkumar, P. (2020). Pyglmnet: Python implementation of elastic-net regularized generalized linear models. *Journal of Open Source Software*, *5*(47), 1959. <https://doi.org/10.21105/joss.01959>

Kim, J.-H. (2009). Estimating classification error rate: Repeated cross-validation, repeated hold-out and bootstrap. *Computational Statistics & Data Analysis*, *53*(11), 3735-3745. <https://doi.org/10.1016/j.csda.2009.04.009>

Montobbio, N., Cavallo, A., Albergo, D., Ansuini, C., Battaglia, F., Podda, J., Nobili, L., Panzeri, S., & Becchio, C. (2022). Intersecting kinematic encoding and readout of intention in autism. *Proceedings of the National Academy of Sciences, USA*, *119*(5), e2114648119. <https://doi.org/10.1073/pnas.2114648119>

Moscatelli, A., Mezzetti, M., & Lacquaniti, F. (2012). Modeling psychophysical data at the population-level: The generalized linear mixed model. *Journal of Vision*, *12*(11), 26. <https://doi.org/10.1167/12.11.26>

Ng, V. K. Y., & Cribbie, R. A. (2016). Using the Gamma Generalized Linear Model for Modeling Continuous, Skewed and Heteroscedastic Outcomes in Psychology. *Current Psychology (New Brunswick, N.J.)*, *36*(2), 225-235. <https://doi.org/10.1007/s12144-015-9404-0>

Patri, J.-F., Cavallo, A., Pullar, K., Soriano, M., Valente, M., Koul, A., Avenanti, A., Panzeri, S., & Becchio, C. (2020). Transient Disruption of the Inferior Parietal Lobule Impairs the Ability to Attribute Intention to Action. *Current Biology*, *30*, 4594-4605. <https://doi.org/10.1016/j.cub.2020.08.104>

Schwarz, G. (1978). Estimating the Dimension of a Model. *The Annals of Statistics*, *6*(2), 461-464. <https://doi.org/10.1214/AOS/1176344136>

Virtanen, P., Gommers, R., Oliphant, T. E., Haberland, M., Reddy, T., Cournapeau, D., Burovski, E., Peterson, P., Weckesser, W., Bright, J., van der Walt, S. J., Brett, M., Wilson, J., Millman, K. J., Mayorov, N., Nelson, A. R. J., Jones, E., Kern, R., Larson, E., . . . van Mulbregt, P. (2020). SciPy 1.0: fundamental algorithms for scientific computing in Python. *Nature Methods*, *17*(3), 261-272. <https://doi.org/10.1038/s41592-019-0686-2>


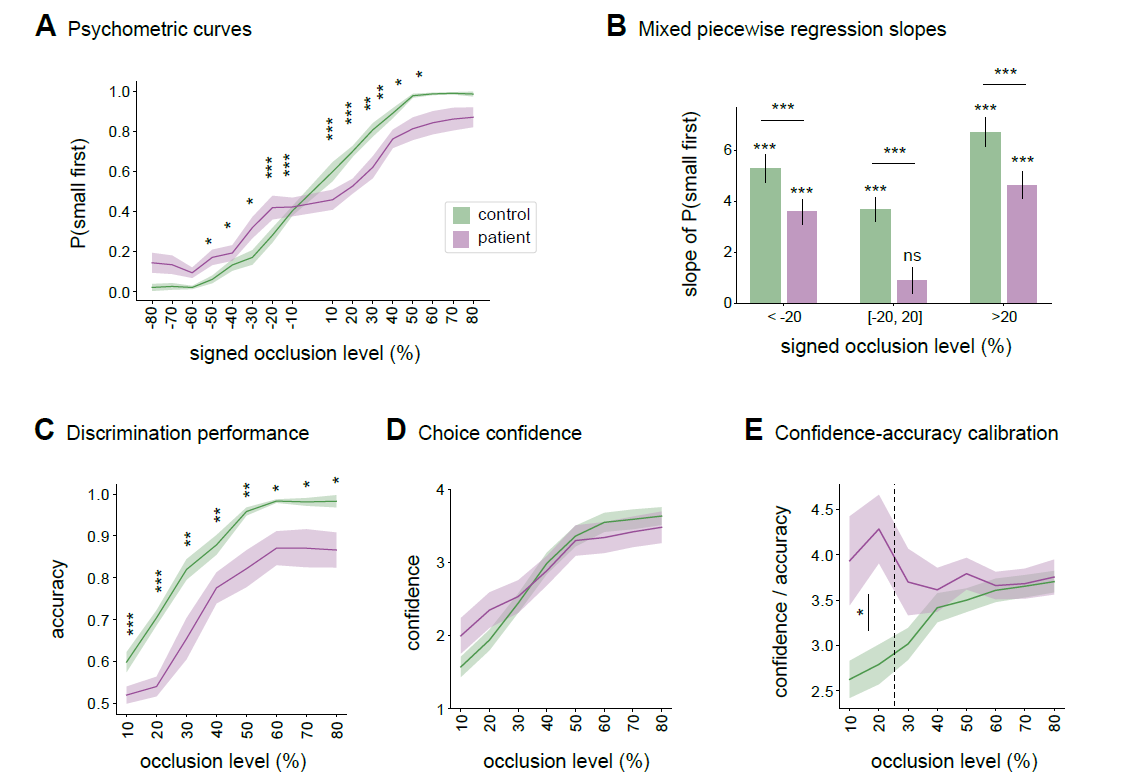


**Supplementary Figure 1.** Results for main analyses including only patients diagnosed with schizophrenia or schizoaffective disorder (N = 14). **(A)** Fitting piecewise regression models to allow for varying slopes of the psychometric curves at different occlusion levels confirmed two integration periods – up to 20% and from 30 to 80%. Replicating the results of the main analyses, the psychometric curve slope **(B)** and the overall prediction accuracy **(C)** of patients with schizophrenia or schizoaffective disorders were close to random during the up-to-20% integration period. As shown in panels **(D-E)**, in patients, the drop in accuracy during this initial period was not accompanied by a decrease in confidence, with the confidence/accuracy ratio being higher for patients than controls. All graphical and statistical conventions are consistent with those used in Figure 1.

**
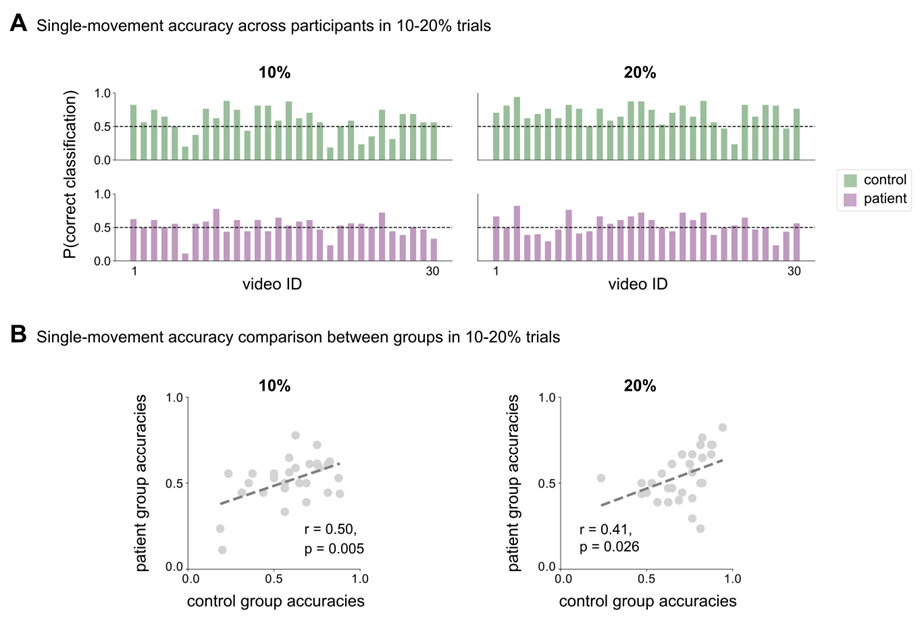
**

**Supplementary Figure 2. Variability of prediction accuracy in the 0-20% period.** *Object size prediction accuracy at the single stimulus level.* Information encoding varies across action stimuli, as does information readout, with prediction accuracies being consistently higher for some action stimuli compared to others. We reasoned that if observers with psychosis extract any size information during the 0-20% period, then they should exhibit a consistent pattern of variation across-trials in this period. Consistent with this prediction, plotting prediction accuracies at the single-stimulus level revealed a distribution that was consistent across different levels of occlusion but varied depending on the specific action stimuli used. **(A)** Bar graphs of single-stimulus prediction accuracy averaged across control observers and observers with psychosis, for 10% (left) and 20% (right) occlusion levels. **(B)** Scatter plot of the relationship between object size prediction accuracy in the patient group and in the control group at 10% and 20% occlusion levels. Data points represent single-stimulus object size prediction accuracy averaged across observers in each group. Fitted regression lines are displayed over the data. Pearson’s correlation coefficients (r) and their significance values (p) are reported. The moderate intergroup correlation, together with single-stimulus prediction accuracies, speaks against a general latency in information.


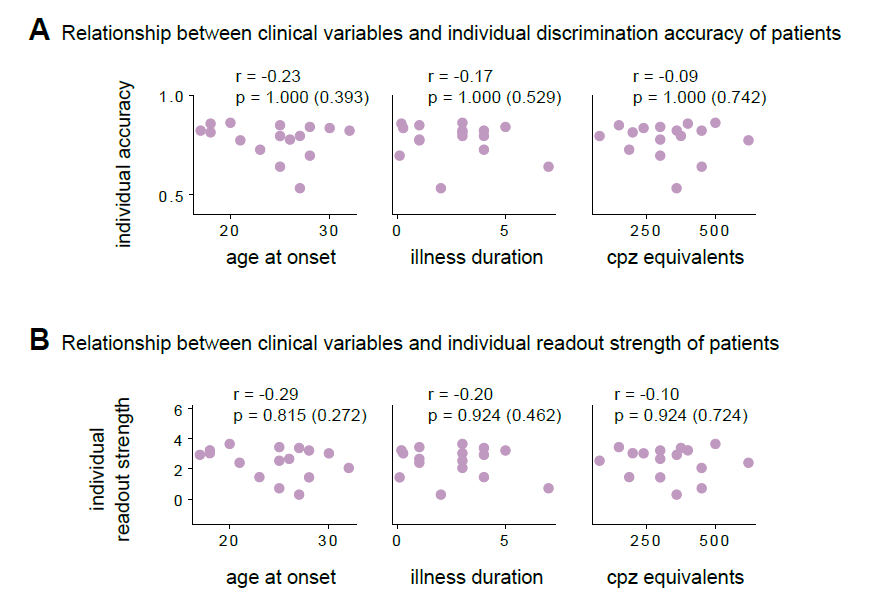


**Supplementary Figure 3.** *Relationship with illness onset, illness duration and antipsychotic medication in patients with psychosis.* Scatter plots of individual prediction accuracy **(A)** and individual readout strength **(B)** of observers with psychosis against illness onset, illness duration and antipsychotic medication. Pearson’s correlation coefficients (r), Holm-Bonferroni corrected significance values (p), and uncorrected significance values (in parentheses) are reported.


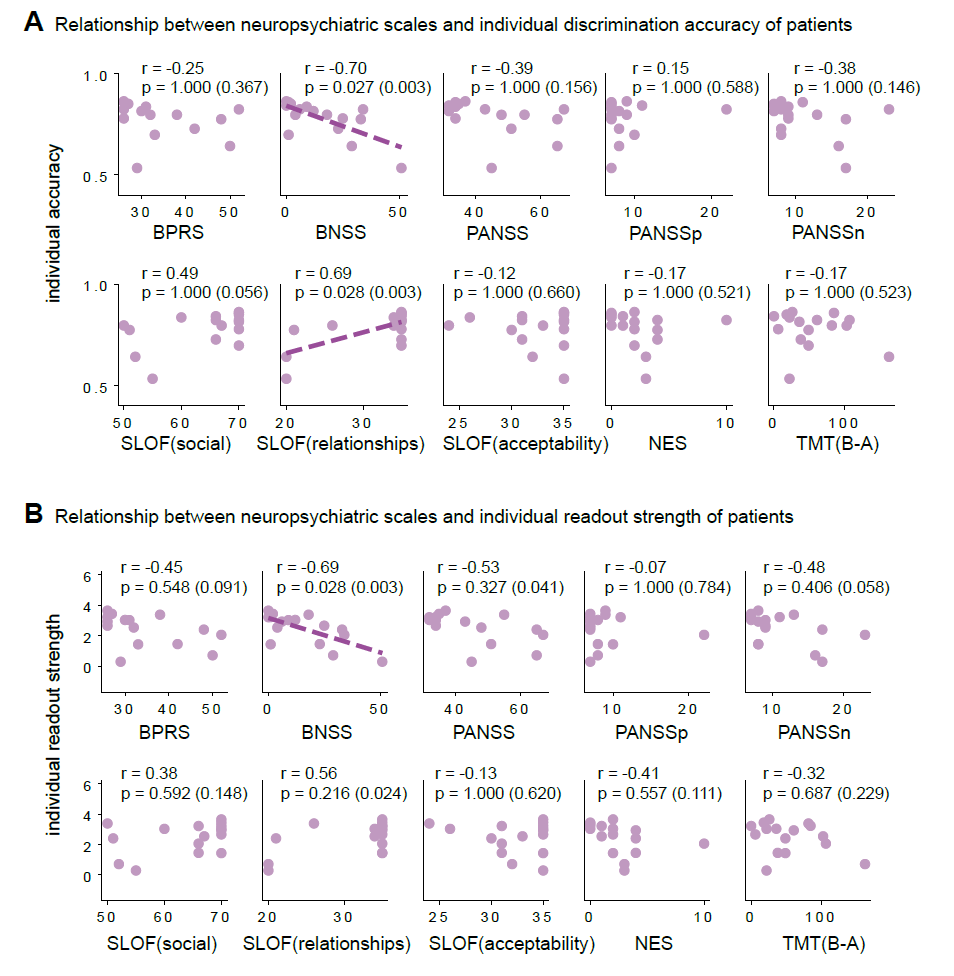


**Supplementary Figure 4.** *Relationship with neuropsychiatric scales in patients with psychosis.* Scatter plots of individual prediction accuracy **(A)** and individual readout strength **(B)** of observers with psychosis against BPRS, BNSS, PANSS, SLOF (social), SLOF (relationships), SLOF (acceptability), NES and TMT (B-A). Pearson’s correlation coefficients (r), Holm-Bonferroni corrected significance values (p), and uncorrected significance values (in parentheses) are reported. For significant linear trends, fitted regression lines are displayed over the data.

**
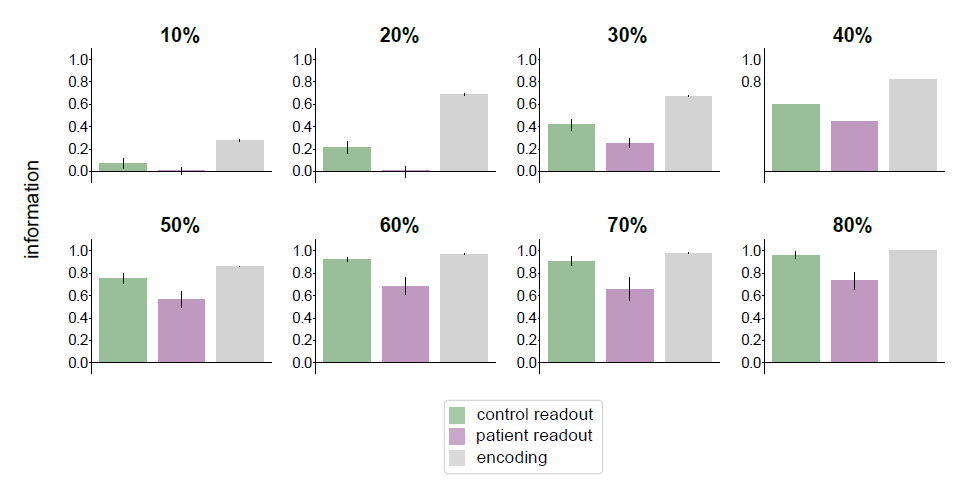
**

**Supplementary Figure 5.** *Information extracted by control participants and patients with psychosis, compared to encoded information across occlusion levels.* We computed the information read by observers in each group (or the information encoded in movement kinematics) as information = (accuracy – chance level) / (1 – chance level) such that 0 corresponds to chance level and 1 corresponds to perfect discrimination. Histograms (mean ± SEM across participants) are presented to compare the information read out by patients and controls against the information encoded at each level of occlusion. To assess the significance of differences in the slope of information encoded and read out across the two integration periods (0-20%, 30-80%), we compared piecewise sigmoidal regression models with non-piecewise models. For information encoding, the non-piecewise sigmoidal model yielded the best fit, with a positive estimated slope of 0.66, 95% CI [0.59,0.80]. For information readout, the piecewise sigmoidal model yielded the best fit for both controls and patients. Controls displayed a significant positive slope already in the initial integration period (estimated slope 0.19, 95% CI [0.14, 0.23]), followed by a substantially higher slope in the second integration period (estimated slope 0.61, 95% CI [0.56, 0.68]). Patients displayed a significant positive slope (estimated slope 0.68; 95% CI [0.65, 0.73]) comparable to that of controls in the second integration period. However, their slope was null in the initial integration period (estimated slope 0.00, 95% CI [0.14, -0.03, 0.02]). These results confirm that controls were already able to extract a significant portion of the encoded information in the initial integration period; in contrast, patients extracted very little if any information up to 30% of movement duration, the gap between patients and controls narrowing progressively from the 30% occlusion level.

|  | | | **Participants with psychosis** | | | | | **Control participants** | |
| --- | --- | --- | --- | --- | --- | --- | --- | --- | --- |
| **Age** | **Sex** | **Medication** | | **Cpz-eq (mg/d)** | **Psychiatric diagnosis** | **Illness onset** | **Illness duration** | **Age** | **Sex** |
| 23 | M | ARI | | 500 | Schizophrenia | 20 | 3 | 31 | F |
| 26 | F | ARI, DEL | | 150 | Schizophrenia | 25 | 1 | 29 | F |
| 27 | M | ARI | | 300 | Schizophrenia | 26 | 1 | 30 | M |
| 29 | M | RIS, SER | | 359 | Schizophrenia | 27 | 2 | 32 | F |
| 28 | M | PRO, RIS, LOR, TRAZ | | 80 | Schizophrenia | 25 | 3 | 30 | M |
| 35 | F | CLO, DEL, LOR, VAL | | 450 | Schizoaffective | 32 | 3 | 34 | F |
| 22 | M | ARI, PRO, MET, MIRT | | 620 | BPD | 21 | 1 | 35 | F |
| 32 | F | OLA, QUE, DEL, DIA | | 450 | Schizoaffective | 25 | 7 | 30 | M |
| 33 | F | ARI, CIT, LA, LOR | | 300 | Schizophrenia | 28 | 5 | 34 | M |
| 31 | M | LUR, CIT, DEL | | 375 | Schizophrenia | 27 | 4 | 35 | M |
| 27 | F | HAL, QUE, LI, LOR | | 187 | Schizophrenia | 23 | 4 | 37 | F |
| 21 | F | ARI, RIS, VAL | | 360 | Schizoaffective | 17 | 4 | 29 | F |
| 18 | M | ARI, SER | | 400 | Schizophrenia | 18 | 0,2 | 24 | F |
| 21 | M | ARI, CIT | | 200 | Schizophrenia | 18 | 3 | 22 | F |
| 28 | F | ARI, DEL | | 300 | Schizophrenia | 28 | 0,1 | 22 | F |
| 30 | M | RIS, CIT | | 240 | BPD | 30 | 0,3 | 26 | F |
| **27 ± 5** |  |  | |  |  |  |  | **30 ± 4** |  |

**Supplementary Table 1.** *Baseline characteristics of participants.* ARI: Aripiprazole; BPD: Brief Psychotic Disorder CIT: Citalopram: CLO: Clozapine; Cpz-eq: Chlorpromazine equivalents; DEL: Delorazepam; DIA: Diazepam; HAL: Haloperidol; LA: Lamotrigine; LI: Lithium; LOR: Lorazepam; LUR: Lurasidone: MET: Methadone; MIRT: Mirtazapine; OLA: Olanzapine; PRO: Promazine; QUE: Quetiapine; RIS: Risperidone; SER: Sertraline; TRAZ: Trazodone; VAL: Valproate.

| **Psychometric curve (probability of reporting small first)** | | | | | |
| --- | --- | --- | --- | --- | --- |
| Random effect structure selection (BIC)  Fixed effects: Observer Group * Occlusion Level, Age, Education | | | | | |
| *Random effect structure* | | *df* | *BIC* | *Deviance* |  |
| Subject (intercept and Occlusion Level slope), Block, Size | | 11 | 5544 | 5446 |  |
| Subject (intercept), Block, Size | | 9 | 5760 | 5679 |  |
| Block, Size | | 8 | 5793 | 5722 |  |
| Subject (intercept and Occlusion Level slope), Size | | 10 | 5572 | 5483 |  |
| Subject (intercept), Size | | 8 | 5784 | 5713 |  |
| Size | | 7 | 5816 | 5754 |  |
| **Subject (intercept and Occlusion Level slope), Block** | | **10** | **5535** | **5446** |  |
| Subject (intercept), Block | | 8 | 5751 | 5679 |  |
| Block | | 7 | 5784 | 5722 |  |
| Subject (intercept and Occlusion Level slope) | | 9 | 5563 | 5483 |  |
| Subject (intercept) | | 7 | 5775 | 5713 |  |
| null | | 6 | 5807 | 5574 |  |
| Fixed effect structure selection (LRT)  Random effects: Subject (intercept and Occlusion Level slope), Block | | | | | |
| *Fixed effect tested* | *df* | | *BIC* | *Deviance* | *p (LRT)* |
| **Observer Group : Occlusion Level** | **10-9** | | **5535-5537** | **5446-5457** | **0.001** |
| Observer Group | **10-9** | | 5535-5529 | 5446-5449 | 0.094 |
| Age | **10-9** | | 5535-5526 | 5446-5446 | 0.989 |
| Education | **10-9** | | 5535-5528 | 5446-5448 | 0.149 |
| Piecewise-sigmoidal fit (BIC) | | | | | |
| *Change points* | *df* | | *BIC* | *Deviance* |  |
| ± 10% | 13 | | 5667 | 5551 |  |
| **± 20%** | **13** | | **5664** | **5548** |  |
| ± 30% | 13 | | 5680 | 5564 |  |
| none | 7 | | 5687 | 5624 |  |
| Selected model  *Fixed effects* Observer Group : Occlusion Level (piecewise-sigmoidal with change points at ±20%)  *Random effects* Subject (intercept and Occlusion Level slope); Block | | | | | |

| **Accuracy** | | | | |
| --- | --- | --- | --- | --- |
| Random effect structure selection (BIC).  Fixed effects: Observer Group * Occlusion Level (piecewise with change point at 20%), Observer Group * Block, Age, Education | | | | |
| *Random effect structure* | *df* | *BIC* | *Deviance* |  |
| **Subject (intercept and Occlusion Level slope)** | **14** | **5811** | **5686** |  |
| Subject (intercept) | 12 | 5908 | 5801 |  |
| null | 11 | 6020 | 5922 |  |
| Fixed effect structure selection (LRT).  Random effects: Subject (intercept and Occlusion Level slope), Block. | | | | |
| *Fixed effect tested* | *df* | *BIC* | *Deviance* | *p (LRT)* |
| **Observer Group : Occlusion Level (piecewise)** | **14-12** | **5811-5821** | **5686-5697** | **0.028** |
| Observer Group : Block | 14-13 | 5811-5802 | 5686-5686 | 0.829 |
| Observer Group | 13-12 | 5802-5793 | 5686-5686 | 0.946 |
| **Block** | **13-12** | **5802-5808** | **5686-5701** | **<0.001** |
| Age | 13-12 | 5802-5794 | 5686-5687 | 0.817 |
| Education | 13-12 | 5802-5787 | 5686-5686 | 0.989 |
| Selected model  *Fixed effects* Observer Group : Occlusion Level (piecewise with change point at 20%); Block.  *Random effects* Subject (intercept and Occlusion Level slope). | | | | |

| **Confidence-accuracy calibration** | | | | |
| --- | --- | --- | --- | --- |
| Random effect structure selection (BIC)  Fixed effects: Observer Group * Integration period, Age, Education | | | | |
| *Random effect structure* | *df* | *BIC* | *Deviance* |  |
| **Subject (intercept and Occlusion Level slope), Block** | **11** | **22941** | **22843** |  |
| Subject (intercept), Block | 9 | 23825 | 23745 |  |
| Block | 8 | 25537 | 25466 |  |
| Subject (intercept and Occlusion Level slope) | 10 | 22969 | 22880 |  |
| Subject (intercept) | 8 | 23843 | 23771 |  |
| null | 7 | 25545 | 25483 |  |
| Fixed effect structure selection (LRT).  Random effects: Subject (intercept and Occlusion Level slope), Block. | | | | |
| *Fixed effect tested* | *df* | *BIC* | *Deviance* | *p (LRT)* |
| **Observer Group : Integration period** | **11-10** | **22941-22986** | **228439-22897** | **<0.001** |
| Age | 11-10 | **22941**-22933 | **228439**-22844 | 0.211 |
| Education | 11-10 | **22941**-22932 | **228439**-22843 |  |
| Selected model  *Fixed effects* Observer Group * Integration period  *Random effects* Subject (intercept and Integration period slope); Block | | | | |

**Supplementary Table 2.** *Generalized Mixed Effects Model.* *Selection of random and fixed effects structure and piecewise-sigmoidal fit* (related to Figure 2)*.* For the backward selection of the fixed structure effects, we conducted likelihood-ratio tests (LRTs) between models differing by one predictor only. We report the tested predictor and the difference in degrees of freedom, BIC and deviance determined by its removal. Tests on effects that are redundant in presence of significant interactions (LRT between equivalent models) are omitted. Retained models and effects are highlighted in bold. The notation A*B indicates both main effects A and B and their interaction (denoted as A:B).

| **Encoding model performance** | | | | |
| --- | --- | --- | --- | --- |
| Random effect structure selection (BIC)  Fixed effects: Occlusion Level | | | | |
| *Random effect structure* | *df* | *BIC* | *Deviance* |  |
| Stimulus (video ID) | 4 | -184.2 | -206.14 |  |
| **null** | **3** | **-189.7** | **-206.14** |  |
| Fixed effect structure selection (LRT).  Random effects: none. | | | | |
| *Fixed effect tested* | *df* | *BIC* | *Deviance* | *p (LRT)* |
| **Occlusion Level** | **3-2** | **-189.7+106.0** | **-206.1+117.0** | **<0.001** |
| Selected model  *Fixed effects* Occlusion Level  *Random effects* - | | | | |

| **Readout model performance** | | | | |
| --- | --- | --- | --- | --- |
| Random effect structure selection (BIC)  Fixed effects: Observer Group * Occlusion Level | | | | |
| *Random effect structure* | *df* | *BIC* | *Deviance* |  |
| Subject (intercept and Occlusion Level slope), Block | 9 | 45791 | 45711 |  |
| **Subject (intercept), Block** | **7** | **45790** | **45728** |  |
| Block | 6 | 45890 | 45836 |  |
| Subject (intercept and Occlusion Level slope) | 8 | 45795 | 45724 |  |
| Subject (intercept) | 6 | 45797 | 45743 |  |
| null | 5 | 45887 | 45843 |  |
| Fixed effect structure selection (LRT)  Random effects: Subject (intercept), Block | | | | |
| *Fixed effect tested* | *df* | *BIC* | *Deviance* | *p (LRT)* |
| Observer Group: Occlusion Level | 7-6 | 45790-45778 | 45728-45724 | 0.837 |
| **Observer Group** | **6-5** | **45778-45775** | **45724-46465** | **0.013** |
| **Occlusion Level** | **6-5** | **45778-46510** | **45724-46465** | **<0.001** |
| Piecewise-linear fit (BIC) | | | | |
| *Change point* | *df* | *BIC* | *Deviance* |  |
| 10% | 7 | 45785 | 45723 |  |
| **20%** | **7** | **45758** | **45695** |  |
| 30% | 7 | 45783 | 45721 |  |
| none | 6 | 45778 | 45724 |  |
| Selected model  *Fixed effects* Observer Group; Occlusion Level (piecewise-linear with change point at 20%)  *Random effects* Subject (intercept and Occlusion Level slope); Block | | | | |

**Supplementary Table 3.** *Linear Mixed Effects Model selection.* *Selection of random and fixed effects structure and piecewise-linear fit* (related to Figures 3B and 4B)*.* For the backward selection of the fixed structure effects, we conducted likelihood-ratio tests (LRTs) between models differing by one predictor only. We report the tested predictor and the difference in degrees of freedom, BIC and deviance determined by its removal. Tests on effects that are redundant in presence of significant interactions (LRT between equivalent models) are omitted. Retained models and effects are highlighted in bold. The notation A*B indicates both main effects A and B and their interaction (denoted as A:B).

| **Object size prediction accuracy** | | | | | | | | | | | | | | | |  |
| --- | --- | --- | --- | --- | --- | --- | --- | --- | --- | --- | --- | --- | --- | --- | --- | --- |
|  | Comparisons against chance (0.5) | | | | | | | | |  | | Comparisons between groups | | | | |
| *Occlusion Level* | Control | | | |  | Patient | | | |  | Control - Patient | | | | |  |
|  | *Est.* | *SE* | *z* | *p* |  | *Est.* | *SE* | *z* | *p* |  | *Diff.* | | *SE* | *z* | *p* |  |
| -80% | **0.007** | **0.003** | **-160.8** | **<0.001**  **(<0.001)** |  | **0.035** | **0.013** | **-34.54** | **<0.001**  **(<0.001)** |  | 0.028 | | 0.013 | 2.141 | 0.059  **(0.032)** |  |
| -70% | **0.013** | **0.005** | **-97.89** | **<0.001**  **(<0.001)** |  | **0.054** | **0.017** | **-25.64** | **<0.001**  **(<0.001)** |  | 0.040 | | 0.017 | 2.331 | 0.059  **(0.020)** |  |
| -60% | **0.025** | **0.008** | **-60.50** | **<0.001**  **(<0.001)** |  | **0.080** | **0.022** | **-19.21** | **<0.001**  **(<0.001)** |  | **0.056** | | **0.022** | **2.523** | **0.049**  **(0.012)** |  |
| -50% | **0.046** | **0.012** | **-37.85** | **<0.001**  **(<0.001)** |  | **0.119** | **0.026** | **-14.39** | **<0.001**  **(<0.001)** |  | **0.073** | | **0.027** | **2.699** | **0.042**  **(0.007)** |  |
| -40% | **0.083** | **0.018** | **-23.65** | **<0.001**  **(<0.001)** |  | **0.172** | **0.031** | **-10.54** | **<0.001**  **(<0.001)** |  | **0.089** | | **0.031** | **2.836** | **0.034**  **(0.005)** |  |
| -30% | **0.147** | **0.025** | **-14.15** | **<0.001**  **(<0.001)** |  | **0.243** | **0.036** | **-7.169** | **<0.001**  **(<0.001)** |  | **0.095** | | **0.033** | **2.923** | **0.031**  **(0.003)** |  |
| -20% | **0.268** | **0.034** | **-6.873** | **<0.001**  **(<0.001)** |  | 0.422 | 0.041 | -1.901 | 0.229  (0.057) |  | **0.154** | | **0.037** | **4.169** | **<0.001**  **(<0.001)** |  |
| -10% | **0.366** | **0.031** | **-4.336** | **<0.001**  **(<0.001)** |  | 0.449 | 0.033 | -1.565 | 0.351  (0.118) |  | **0.083** | | **0.020** | **4.177** | **<0.001**  **(<0.001)** |  |
| 10% | **0.589** | **0.030** | **2.950** | **0.003**  **(0.003)** |  | 0.504 | 0.031 | 0.125 | 0.901  (0.901) |  | **-0.085** | | **0.020** | **-4.188** | **<0.001**  **(<0.001)** |  |
| 20% | **0.693** | **0.034** | **5.721** | **<0.001**  **(<0.001)** |  | 0.531 | 0.039 | 0.805 | 0.841  (0.421) |  | **-0.162** | | **0.038** | **-4.215** | **<0.001**  **(<0.001)** |  |
| 30% | **0.835** | **0.026** | **13.07** | **<0.001**  **(<0.001)** |  | **0.716** | **0.037** | **5.780** | **<0.001**  **(<0.001)** |  | **-0.120** | | **0.035** | **-3.462** | **0.006**  **(0.001)** |  |
| 40% | **0.921** | **0.015** | **27.28** | **<0.001**  **(<0.001)** |  | **0.822** | **0.029** | **11.05** | **<0.001**  **(<0.001)** |  | **-0.100** | | **0.030** | **-3.331** | **0.010**  **(0.001)** |  |
| 50% | **0.964** | **0.009** | **52.44** | **<0.001**  **(<0.001)** |  | **0.894** | **0.022** | **18.02** | **<0.001**  **(<0.001)** |  | **-0.070** | | **0.023** | **-3.124** | **0.018**  **(0.002)** |  |
| 60% | **0.984** | **0.005** | **100.0** | **<0.001**  **(<0.001)** |  | **0.939** | **0.016** | **28.04** | **<0.001**  **(<0.001)** |  | **-0.045** | | **0.016** | **-2.858** | **0.034**  **(0.004)** |  |
| 70% | **0.993** | **0.003** | **193.6** | **<0.001**  **(<0.001)** |  | **0.966** | **0.011** | **43.53** | **<0.001**  **(<0.001)** |  | **-0.027** | | **0.011** | **-2.579** | **0.049**  **(0.010)** |  |
| 80% | **0.997** | **0.001** | **382.0** | **<0.001**  **(<0.001)** |  | **0.981** | **0.007** | **68.32** | **<0.001**  **(<0.001)** |  | -0.016 | | 0.007 | -2.318 | 0.059  **(0.020)** |  |

| **Piecewise regression slopes of the psychometric curves** | | | | | | | | | | | | | | |  |
| --- | --- | --- | --- | --- | --- | --- | --- | --- | --- | --- | --- | --- | --- | --- | --- |
|  | Comparisons against 0 | | | | | | | | |  | Comparisons between groups | | | |  |
| *Integration period* | Control | | | |  | Patient | | | |  | Control - Patient | | | |  |
|  | *Slope* | *SE* | *z* | *p* |  | *Slope* | *SE* | *z* | *p* |  | *Slope difference* | *SE* | *z* | *p* | |
| < - 20% | **5.112** | **0.526** | **9.716** | **<0.001**  **(<0.001)** |  | **3.462** | **0.469** | **7.387** | **<0.001**  **(<0.001)** |  | **-1.65** | **0.540** | **-3.054** | **0.002**  **(0.002)** | |
| [-20%, 20%] | **3.642** | **0.479** | **7.598** | **<0.001**  **(<0.001)** |  | 0.883 | 0.463 | 1.905 | 0.057  (0.057) |  | **-2.76** | **0.661** | **-4.171** | **<0.001**  **(<0.001)** | |
| > 20% | **6.702** | **0.576** | **11.634** | **<0.001**  **(<0.001)** |  | **4.834** | **0.508** | **9.513** | **<0.001**  **(<0.001)** |  | **-1.87** | **0.518** | **-3.604** | **<0.001**  **(<0.001)** | |

| **Confidence-accuracy calibration** | | | | | | | | | | | |  |
| --- | --- | --- | --- | --- | --- | --- | --- | --- | --- | --- | --- | --- |
| Comparisons between groups | | | | | | | | | | | |  |
|  | 10-20% integration period | | | |  | | 30-80% integration period | | | | |  |
|  | *Estimate* | *SE* | *z* | *p* | |  | | *Estimate* | *SE* | *z* | *p* | |
| Control - Patient | **-0.592** | **0.297** | **-1.991** | **0.047** | |  | | 0.073 | 0.281 | 0.261 | 0.794 | |

| **Effect of block on accuracy across groups** | | | |
| --- | --- | --- | --- |
| Comparison against 0 | | | |
| *Slope* | *SE* | *z* | *p* |
| **0.458** | **0.115** | **3.977** | **<0.001** |

**Supplementary Table 4.** *Coefficient analysis for the retained Generalized Mixed Effects for object size prediction accuracy, piecewise regression slopes of the psychometric curves, confidence-accuracy calibration and effect of block on accuracy* (related to Figure 2)*.* For all comparisons, we report the model estimate and standard error (SE) of the tested quantity, the *z*-value and the two-sided *p*-value computed from the z-test. All *p*-values are Holm-Bonferroni corrected for the number of comparisons listed for each entry, and uncorrected *p*-values are reported in parentheses. Significant comparisons are highlighted in bold.

| **Encoding model performance** | | | | |
| --- | --- | --- | --- | --- |
| Comparisons against chance (0.5) | | | | |
| *Occlusion Level* | *Estimate* | *SE* | *t* | *p* |
| 10% | **0.733** | **0.019** | **12.489** | **<0.001 (<0.001)** |
| 20% | **0.779** | **0.015** | **18.458** | **<0.001 (<0.001)** |
| 30% | **0.825** | **0.012** | **26.636** | **<0.001 (<0.001)** |
| 40% | **0.871** | **0.010** | **35.517** | **<0.001 (<0.001)** |
| 50% | **0.917** | **0.010** | **39.930** | **<0.001 (<0.001)** |
| 60% | **0.963** | **0.012** | **37.973** | **<0.001 (<0.001)** |
| 70% | **1.009** | **0.015** | **33.718** | **<0.001 (<0.001)** |
| 80% | **1.056** | **0.019** | **29.807** | **<0.001 (<0.001)** |

| **Readout model performance (*z*-scored by permutation distribution)** | | | | | | | | | |
| --- | --- | --- | --- | --- | --- | --- | --- | --- | --- |
| Comparisons against 0 | | | | | | | | | |
| *Occlusion Level* | Control group | | | |  | Patient group | | | |
|  | *Estimate* | *SE* | *z* | *p* |  | *Estimate* | *SE* | *z* | *p* |
| 10% | **0.884** | **0.246** | **3.594** | **<0.001**  **(<0.001)** |  | 0.150 | 0.246 | 0.610 | 0.542  (0.271) |
| 20% | **0.867** | **0.268** | **3.238** | **<0.001**  **(<0.001)** |  | 0.133 | 0.268 | 0.496 | 0.310  (0.310) |
| 30% | **2.609** | **0.244** | **10.68** | **<0.001**  **(<0.001)** |  | **1.875** | **0.244** | **7.675** | **<0.001**  **(<0.001)** |
| 40% | **3.178** | **0.236** | **13.46** | **<0.001**  **(<0.001)** |  | **2.444** | **0.236** | **10.35** | **<0.001**  **(<0.001)** |
| 50% | **3.749** | **0.232** | **16.14** | **<0.001**  **(<0.001)** |  | **3.013** | **0.232** | **12.98** | **<0.001**  **(<0.001)** |
| 60% | **4.316** | **0.233** | **18.53** | **<0.001**  **(<0.001)** |  | **3.582** | **0.233** | **15.37** | **<0.001**  **(<0.001)** |
| 70% | **4.885** | **0.238** | **20.50** | **<0.001**  **(<0.001)** |  | **4.151** | **0.238** | **17.41** | **<0.001**  **(<0.001)** |
| 80% | **5.454** | **0.248** | **22.00** | **<0.001**  **(<0.001)** |  | **4.720** | **0.248** | **19.03** | **<0.001**  **(<0.001)** |

**Supplementary Table 5.** *Coefficient analysis for the retained Linear Mixed Effects Models for encoding and readout model performance* (related to Figures 3B and 4B)*.* For all comparisons, we report the model estimate and standard error (SE) of the tested quantity, the *t*-value or *z*-value and the two-sided *p*-value computed from the corresponding test. All *p*-values are Holm-Bonferroni corrected for the number of comparisons listed for each entry, and uncorrected *p*-values are reported in parentheses. Significant comparisons are highlighted in bold.
